# Hepatitis B infection awareness, vaccine perceptions and uptake, and serological profile of a group of health care workers in Yaoundé, Cameroon

**DOI:** 10.1186/s12889-016-3388-z

**Published:** 2016-08-03

**Authors:** Henri Olivier Pambou Tatsilong, Jean Jacques N. Noubiap, Jobert Richie N. Nansseu, Leopold N. Aminde, Jean Joel R. Bigna, Valentine Ngum Ndze, Roger Somo Moyou

**Affiliations:** 1 Mvog-Ada Health Center, Djoungolo Health District, Yaoundé, Cameroon; 2 Higher Institute of Health Professions, Faculty of Sciences, University of Ngaoundéré, Ngaoundéré, Cameroon; 3 Department of Medicine, Groote Schuur Hospital and University of Cape Town, 7295 Cape Town, South Africa; 4 Medical Diagnostic Center, Yaoundé, Cameroon; 5 Department of Public Health, Faculty of Medicine and Biomedical Sciences, University of Yaoundé 1, Yaoundé, Cameroon; 6 Clinical Research Education, Networking and Consultancy (CRENC), Douala, Cameroon; 7 School of Public Health, The University of Queensland, QLD 4006 Brisbane, Australia; 8 Department of Epidemiology and Public Health, Pasteur Center of Cameroon, Yaoundé, Cameroon; 9 Centre for Evidence-based Health Care, Faculty of Medicine and Health Sciences, Stellenbosch University, Cape Town, South Africa; 10 Faculty of Medicine and Biomedical Sciences, University of Yaoundé 1, Yaoundé, Cameroon

**Keywords:** Viral hepatitis B, Health care workers, Knowledge, Vaccine, Serological markers

## Abstract

**Background:**

Cameroon is one of the countries in Africa with the highest burden of Hepatitis B infection. Health care workers are known to be at risk of occupational exposure to blood and other infectious bodily fluids. The aim of this study was to assess the profile of serological markers of hepatitis B virus (HBV) infection, knowledge and perceptions regarding HBV infection among health care workers in a health area in Yaoundé.

**Methods:**

A cross-sectional study was conducted in the Mvog-Ada Health Area of the Djoungolo Health District from March 1 to November 31, 2014. All consenting health care workers were included in the study. Serological markers of HBV (HBs Ag, Hbe Ag, anti-HBs Ab, anti-HBe Ab, anti-HBc Ab) were qualitatively tested using Biotech®(OneHBV-5 parameter rapid test website) in each participant and the anti-HBs antibodies were quantified by ELISA (Biorex) among those who were positive with the qualitative test. Chi square test or its equivalents were used to compare qualitative variables and a *p*-value less than or equal to 0.05 was considered significant.

**Result:**

A total of 100 participants were retained for the study out of 163 in the health area giving a response rate of 61.34 %; the mean age was 30.5 (SD 6.8) years and 71 % of participants were women. Forty seven percent (47 %) of workers had good level of knowledge of HBV infection. The men were 3.20 times (95 % CI: 1.02–9.19, *p* = 0.04) more likely to have a good level of knowledge than women. Participants with a university study level were more (95 % CI: 3.17–25, *p* < 0.0001) likely to have a good level of knowledge than those with a high school study level. Ninety-six percent of participants thought that they were at a greater risk of becoming infected with HBV than the general population, 93 % felt that the vaccine should be compulsory and all (100 %) were willing to recommend it to others. However, only 19 % had received at least one dose of the vaccine. The proportion of HBs Ag was 11 %. The different serological profiles with regard to HBV infection were naive subjects (62 %), chronic carriers (11 %), vaccinated (19 %) and subjects naturally immunized (8 %). Three out of the 19 participants who received at least one dose of the vaccine, only 9 (47.4 %) of whom had titers ≥100 IU/l indicating a good response to vaccination. Among those who received three doses of the vaccine (*n* = 12, 63 %), 2 (16, 66 %) had poor response to vaccination (HBs Ab titers < 100 IU/l).

**Conclusion:**

The prevalence of HBs Ag among health care workers in the Mvog-Ada Health Area is high (11 %). These workers are at high risk of HBV infection because of very low vaccine uptake and poor post-exposure practices. Their knowledge of HBV infection is non-optimal.

## Background

Hepatitis B virus (HBV) infection is a major health problem worldwide owing to its high prevalence and significant morbidity and mortality. According to a recent systematic review and meta-analysis estimating the worldwide prevalence of chronic HBV infection, in 2010, about 248 million individuals were hepatitis B surface antigen (HBs Ag) positive [1]. More than 780 000 people die every year due to hepatitis B-related complications including cirrhosis and hepatocellular carcinoma [2]. The prevalence of HBV chronic infection is particularly high in Africa, prevalence reaching up to 22 % in South Sudan [1]. In Cameroon, seroprevalence of HBs Ag has been reported to be 10.1 % in a general population of blood donors [3], 10.2 % among pregnant women [4] and 23.7 % among HIV-infected patients [5].

Health care workers are at a high risk of HBV infection through occupational exposure to blood, and the incidence of this infection among them has been estimated to be 2–4 times the level in the general population [6]. As part of occupational safety measures, it has been recommended that all health care workers should be vaccinated against HBV [7]. In fact, HBV infection is preventable for more than twenty years through effective prophylactic vaccination [8]. But intriguingly, WHO has reported that HBV vaccination coverage amongst health care workers is only 18–39 % in low–and middle–income countries (LMIC) compared to 67–79 % in high-income countries [9].

In Cameroon, few studies have been done on the epidemiology of HBV among health care providers. We previously conducted two studies among surgical residents [10] and medical students [11] in Cameroon which showed that despite a good knowledge of the risk factors for HBV infection and awareness of HBV vaccine, only 24.5 % and 18 % of these respective populations were adequately vaccinated against HBV. The main limitation of these studies was the absence of serological testing to determine the prevalence of HBV infection and the immune status of participants. We therefore conducted the current study which aimed to evaluate knowledge of HBV infection, HBV vaccine uptake, and serological profile of a group of health care workers in Yaoundé, Cameroon.

## Methods

### Study design, Setting and population

This was a cross-sectional study conducted from March to November 2014 in the Mvog-Ada Health Area, which is part of the Djoungolo Health District, one of the eight health districts of Yaoundé, the capital city of Cameroon. This health area covers an average population of 82,000 inhabitants and comprises six health facilities with a total of 163 health care workers during the study period.

Participants were health care providers working in one of the health centers of Mvog-Ada Health Area, irrespective of their age, sex, background or seniority, who were at their post when the investigator visited, and who voluntarily accepted to take part in the study. The sample size was calculated, considering a 6.6 % prevalence of HBs Ag carriage among health care workers as reported by Noah et al. [12] in Yaoundé, and a 5 % level of precision; hence a minimum of 96 subjects was required.

### Data collection and laboratory investigation

A structured pretested questionnaire was used to collect socio-demographic information (age, sex, qualification and year experience) and data on risk factors for HBV infection among participants (practice and attitude). Upon completion of the questionnaire, 5 ml of venous blood was aseptically collected by venipuncture into an Ethylene Di-amine Tetra-acetic Acid (EDTA) tube. The plasma obtained from each sample was tested for serological markers of HBV including HBs Ag, hepatitis B e antigen (Hbe Ag), hepatitis B surface antibodies (anti-HBs Ab), hepatitis B e antibodies (anti-HBe Ab), and hepatitis B core antibodies (anti-HBc Ab). These tests were performed using a qualitative test Biotech® (OneHBV-5 parameter rapid test website, CTK Biotech, Inc. 6748 Nancy Ridge Drive, San Diego, CA 92121, USA) in each participant, and the anti-HBs Ab were further quantified by ELISA (Biorex Diagnostics Limited, Unit 2C Antrim Technology Park, Muckamore; United Kingdom) among those who were positive with the qualitative test.

### Assessment of knowledge on HBV and HBV vaccination

The level of knowledge of participants on HBV and HBV vaccination was estimated using a score based on their responses to 13 questions. Each corrected answer was allocated 1 point, giving a total score of 13 points. A good level of knowledge was considered as at least 10/13. This minimum of 10/13 defining a good level of knowledge may seem rigorous. However, this minimum level justified an affordable difficulty of the questions.

### Serological profile and immune status

The following definitions were applied [13]:

**Table:** 

	HBs Ag	HBe Ag	HBs Ab	HBe Ab	HBc Ab
Naive subjects	-	-	-	-	-
Chronic carriers	+	-	-	+	+
Subjects vaccinated	-	-	+	-	-
Subjects naturally immunized	-	-	+	-	+
Acute hepatitis	+	+	-	+	-

+: positive - : negative

Regarding immune status, participants with a HBs Ab titer under 10 IU/L were considered unimmunized, those with a titer between 10 IU/L and 100 IU/L were considered being poorly immunized and those with a titer equal or greater than 100 IU/L had good immunity against HBV [14].

### Data analysis

Data were coded, entered and analyzed using IBM SPSS software (Version 20, Chicago, Illinois, USA). Results are presented as counts (proportions) for categorical variables, and means with standard deviations (SD) for quantitative ones. Odds ratios (OR) with their 95 % confidence intervals (CI) served to investigate the influence of various factors on the occurrence of HBV infection, and were calculated by both univariate and multivariate logistic regression analyses while adjusting for potential confounders. We included in the multivariate model the age and all variables with a *p* value ≤ 0.2 in univariate analyses. For this purpose, we categorized some variables into two groups. The age was grouped into two classes: ≤ 30 and > 30 years. Educational level was classified as ‘low’ for participant having a secondary level of education or less, and ‘high’ for those with a University level of education or more. Specialty was categorized as Laboratory for “Laboratory technician, Assistant laboratory technician” and Medicine for “Doctor, Nurse and Midwife”. The professional experience was dichotomized in two groups: ≤ 6 years and > 6 years. The cut-off of 6 years was chosen because it represents the median duration of work experience among our participants. A *p* value < 0.05 was set to characterize statistically significant results.

## Results

### General characteristics

Of the 163 health care workers in the health area, 100 were included in the study giving a participation rate of 61.34 %. The mean age was 30.5 (SD 6.8) years and the male/female sex ratio was 0.40. We enrolled three doctors (3 %), seven midwifes (7 %), 13 laboratory technicians (13 %), 21 nurses (21 %), 28 nurse aids (28 %), 11 assistant laboratory technicians (11 %) and 17 auxiliary staffs (17 %) (Table 1).

**Table 1:** Distribution of participants according to age, sex, level of education and number of years of service

Characteristics	Number or percentage^a^
Age	
20–29	43
30–39	44
40–55	14
Sex	
Male	29
Female	71
Level of education	
Secondary	53
University	47
Qualification	
Nurse aids	28
Lab technician	13
Assistant lab technician	11
Nurse	21
Midwife	7
Doctor	3
Auxiliary staff	17
Service	
Laboratory	28
Maternity	19
Surgery	7
Medicine	46
Professional experience	
<6	60
≥6	40

^a^Total enrollment is 100, the number of the group are identical to the percentages

### Level of knowledge on HBV and correlates

Forty seven participants (47 %) had a right level of knowledge of HBV infection. Only 26 % of participants were aware that HBV is the most contagious blood-borne virus. Most of participants correctly identified injury with contaminated needle (96 %), sexual intercourses (96 %), mother-to-child transmission (96 %) and blood transfusion (99 %) as routes of contamination with HBV. All of them were aware of the existence of a vaccine to prevent HBV infection, up to 77 % knew that the minimum number of doses for a complete primary HBV vaccination is 3 doses, but only 57 % knew that an immune response test should be done after HBV vaccination to confirm a good immunization status (Table 2). As shown in Table 3, in multivariate analysis, men were 3.20 times more likely to have a good level of knowledge than women (95 % CI: 1.02 – 9.19, *p* = 0.04). Participants with a high educational level were 8.85 times more likely to have a good level of knowledge than those with at most a high school study level (95 % CI: 3.17-25, *p* < 0.0001).

**Table 2:** Knowledge of risk factors and HBV vaccination among 100 health workers in Cameroon

Statements	Correct answers Frequency or percentage
1. HBV is the most contagious blood-borne pathogen through accidental exposure to blood	26
2. Contact of healthy skin with infected blood is a risk factor of HBV infection	46
3. Injury with needle contaminated with infected blood is a risk factor of HBV infection	96
4. Contact of abraded skin with infected body fluid is a risk factor of HBV infection	81
5. Contact of eyes with infected blood is a risk factor of HBV infection	69
6. HBV could be transmitted through sexual intercourse	96
7. HBV could be transmitted through oro-fecal route	44
8. HBV could be transmitted through blood	99
9. HBV could be transmitted from a mother to his foetus	96
10. Immunoglobulin against HBV can prevent infection after exposure	39
11. There is a vaccine available against HBV	100
12. The minimum number of doses for a complete primary HBV vaccination is 3 doses	77
13. An immune response test should be done after HBV vaccination	57

*HBV* hepatitis B virus

**Table 3:** Unadjusted and adjusted factors associated with good knowledge of HBV infection

Variable	Total	Good knowledge n (%)	OR (95 % CI)	*p* value	aOR (95 % CI)	*p* value
Age						
≤30	50	22 (44)	0.46 (0.15–1.54)	0.21	0.43 (0.16–1.2)	0.10
>30	50	25 (50)	1		1	
Sex						
Male	29	22 (75.86)	3.44 (1.075–1.032)	0.03	3.19 (1.02–9.91)	0.04
Female	71	25 (35.21)	1		1	
Level of education						
University	47	35 (74.46)	8.20 (2.87–23.81)	<0.0001	8.85 (3.17–25)	<0.0001
Secondary	53	12 (22.64)	1		1	
Service						
Laboratory	28	15 (53.57)	3.70 (0.32–42.67)	0.29		
Medicine	72	32 (44.44)	1			
Professional experience						
<6 years	60	30 (50)	0.85 (025–285)	0.79		
≥6 years	40	17 (42.50)	1			

*OR* odds ratio, *aOR* adjusted odds ratio, *CI* confidence interval

The variables introduced in the multivariable model were age, sex and educational level

### Perception of HBV vaccine, vaccination status and history of exposure to blood

Ninety-six percent of participants thought that they were at a greater risk of becoming infected with HBV than the general population and 93 % felt that vaccination against HBV should be compulsory for all health care workers and all of them (100 %) were willing to recommend it to others colleagues. While 67 % of participants considered the HBV vaccine as safe, only 19 % had received at least one dose of the vaccine. Among those who never had any dose of the HBV vaccine (*n* = 81), the reasons for not being vaccinated were lack of sufficient information on the vaccine (49.4 %, *n* = 40), lack of money to pay for the vaccine (33.3 %, *n* = 27), lack of time (12.3 %, *n* = 10) and lack of motivation (4.9 %, *n* = 4). Moreover, 41 % of participants had been informed by their training institution or by the administration of their health facility about the importance of being vaccinated against HBV. All unvaccinated participants were willing to receive the HBV vaccine.

Regarding history of accidental exposure, while 64 % of participants reported occupational exposure to HBV in the past, only 35.9 % (*n* = 23) of them had always notified the exposure and 75 % (*n* = 48) never considered the risk of HBV infection after exposure but only the risk of HIV infection.

### Serological profile

The frequency of HBs Ag was 11 %, while that of HBs Ab was 26 % (Table 4). Sixty-two percent of subjects were naive to HBV and mostly females (74.2 %, *n* = 46). Those working outside the laboratory (79 %, *n* = 49) and those with less than 6 years of professional experience (64.5 %, *n* = 40). Nineteen percent were vaccinated, mostly those working outside the laboratory (73.7 %, *n* = 14), females (63.2 %, *n* = 12) and those with less than 6 years of professional experience (63.2 %, *n* = 12). Eight percent were naturally immunized and 11 % chronic carriers (Table 5).

**Table 4:** Distribution of following serological markers according to the qualification

Qualification	HBs Ag positive	HBs Ab positive	HBe Ag positive	HBe Ab positive	HBc Ab positive	Total
Nurse aid	3 (10.7)	6 (21.4)	0 (0)	3 (10.7)	5 (17.9)	28
Assistant Lab technician	2 (18.1)	2 (18.1)	0 (0)	2 (18.1)	2 (18.1)	11
Lan technician	4 (30.8)	5 (38.5)	0 (0)	5 (38.5)	8 (61.5)	13
Nurse	2 (9.5)	6 (28.6)	1 (4.8)	1 (4.8)	3 (14.3)	21
Doctor	0 (0)	1 (33.3)	0 (0)	0 (0)	0 (0)	3
Midwife	0 (0)	2 (28.6)	0 (0)	0 (0)	0 (0)	7
Auxiliary staff	0 (0)	4 (23.5)	0 (0)	1 (5.9)	0 (0)	17
Total	11 (11)	26 (26)	1 (100)	12 (12)	18 (18)	100

**Table 5:** Hepatitis B serological profiles according to age, sex, level of education, service and experience

Characteristics	Naive	Vaccinated	Naturally immunized	Chronic carrier	Total
Age					
≤30	33	8	1	8	50
>30	29	11	7	3	50
Sex					
Male	16	7	1	5	29
Female	46	12	7	6	71
Educational level					
University	28	9	4	6	47
Secondary	34	10	4	5	53
Service					
Laboratory	13	5	4	6	28
Medicine	49	14	4	5	72
Experience					
<6 years	40	12	1	7	60
≥6 years	22	7	7	4	40
Total	62	19	8	11	100

Serologically immunized participants (19 %) where all those who reported having received at least one dose of HBV vaccine. Among unvaccinated participants; 8.6 % had the anti-Hbs ab, anti-HBc Ab and anti- Hbe Ab; these markers reflect a natural immunity. We quantified HBs Ab and titers ranged from 0 IU/l to 251 IU/l. Three out of the 19 participants who were vaccinated (15.8 %) had HBs Ab titers <10 IU/l, while 7 (36.8 %) had titers ≥10 IU/l and < 100 IU/l, and 9 (47.4 %) had titers ≥100 IU/l indicating a good response to vaccination. Among those who received three doses of the vaccine (*n* = 9), 2 had poor response to vaccination (HBs Ab titers < 100 IU/l).

## Discussion

The current study was designed to assess knowledge of HBV infection, HBV vaccine perception and uptake, and determine the serological profile of a group of health care workers in Yaoundé, Cameroon. Forty-seven percent of the participants had a good level of knowledge on HBV infection. After adjusting for confounders, correlates of a good level of knowledge included male gender and high educational level. The uptake of HBV vaccine was very low (19 %). Serological testing showed that 62 % of participants were naïve to HBV, 10 % were chronic carriers. Among the 19 participants who received at least one dose of the vaccine, only 9 had a good response to vaccination.

In two previous studies, we reported good knowledge about HBV infection among medical students [11] and surgical residents [10], contrasting with the findings of the current study which revealed that only 47 % of health care workers had a good level of knowledge. This difference is most likely due to the fact that participants with high educational level were about 9 times more prone to have a right level of knowledge as had surgical residents or medical students in these two previous studies, and these participants with high educational level represented only 47 % of the study population. This low level of knowledge is however similar to that reported among health care workers in Northwest Ethiopia, where only 52 % of the respondents were knowledgeable about hepatitis B infection [15].

Almost all participants (96 %) recognized that health care workers are more at risk of being infected with HBV than the general population, and over two-thirds of participants considered that the vaccine against HBV is safe. Similarly, among surgical residents and medical students in Cameroon, we found that respectively 78.4 % of participants and 97.3 % thought that the HBV vaccine was safe and 87.4 %, and 78.4 % recognized that HCW were more at risk [10, 11]. Our participants felt that vaccination should be mandatory for health care providers (93 %) and all (100 %) participants agreed to recommend HBV vaccination to their colleague. However, only 19 % were vaccinated.

Similar low uptake of HBV vaccine was reported in our previous studies among surgical residents (24.5 %) [10] and medical students (18 %) [11], and in another study among health care workers in Yaoundé (8.8 %) [12]. Among health profession trainees, HBV vaccine coverage was reported to be 47.7 % among medical students [16] and 37.9 % among dental students in Nigeria [17], 76.8 % among medical students and 46.7 % among interns in Palestine [18]. A study among theatre personnel in Nigeria reported a HBV uptake rate of 26.8 % [19], while 5.4 % and 52 % of health care workers respectively in Northwest Ethiopia [15] and Libya [20] reported that they took three or more doses of HBV vaccine.

Studies have shown that awareness of risk among health care workers is an important factor affecting HBV vaccine uptake [21–23]. Contrariwise, the low vaccine coverage among our participants contrasts with the fact that almost all of them (96 %) considered that they are at a higher risk of HBV infection than the general population. The main reason of this low vaccine uptake was reported to be lack of sufficient information on the vaccine. This reason is in keeping with the fact that only 41 % of participants had been informed by their training institution or by the administration of their health facility about the importance of being vaccinated against HBV. Therefore, raising the awareness of health care workers about the importance of being vaccinated against HBV and providing them with relevant information about HBV vaccine is paramount to scale up vaccine uptake in this population at high risk of HBV infection.

Another important reason of not being vaccinated was lack of money to pay for the vaccine. This stresses the need to subsidize HBV vaccination among health care workers. Considering the fact that HBV vaccine is freely provided to children inside the national Expanded Programme for immunization (EPI) since 2005, our health care system should afford subsidizing the vaccine for health care workers bearing in mind their high risk of being contaminated by or to contaminate their patients. Indeed, there is an urgent need for health policies advocating HBV vaccination among health care workers in Cameroon in order to curb the burden and occupational risk of HBV infection in the hospital environment.

Furthermore, we found that only 9 out of the 19 participants who were vaccinated had HBs Ab titers ≥100 IU/l indicating a good response to vaccination. In fact, only 9 % of our study population was adequately vaccinated and thus protected against HBV infection. Among the 9 participants who said they took three doses of the vaccine, 2 had poor response to vaccination (HBs Ab titers < 100 IU/l), highlighting the necessity to do an immune response test after the 3 doses of the minimum HBV vaccination course, in order to have a booster dose in case of poor immune response.

The prevalence of HBs Ag in our study population was 11 %, with 10 % chronic carriers and 1 % of active HBV infection. These health care workers may develop fatal complications such as cirrhosis and hepatocellular carcinoma. These workers can also potentially transmit HBV to their patients. In Cameroon, seroprevalence of HBs Ag has been reported to be 10.1 % in a general population of blood donors [3], 10.2 % among pregnant women [4] and 23.7 % among HIV-infected patients [5]. Among health care workers, a study in Yaoundé reported a prevalence of 6.6 % lower than what we found [12]. Among health care workers the rate of HBs Ag positivity was 1.8 % in a study in Libya [20], 2.9 % in Rwanda [24], 7 % in Tanzania [25] and 8.1 % in Uganda [26]. The higher frequency of HBV infection in our study as compared to those from other countries is mainly due to the high prevalence of the infection in the general population.

Finally, 62 % of our participants had never been exposed to HBV and therefore susceptible or at risk of HBV infection. These subjects highly need vaccination to be protected. The importance of vaccination is highlighted by the very poor preventive practices of participants after exposure to blood. Indeed, those exposed did not notify the exposure to the authorities in their health facilities, to seek post-exposure prophylaxis. Moreover, the majority (75 %) initially only considered the risk of HIV infection and only 25 % considered that of HBV infection. This could be due to the fact that, in most public health awareness campaigns, a lot of emphasis is made on HIV/AIDS, leading to greater consciousness on HIV in the general population and among health care workers as opposed to HBV despite its high infectivity. While it is important to improve attitudes towards post-exposure prophylaxis, vaccination against HBV remains overriding.

## Conclusion

Health care workers in the Mvog-Ada Health Area are at high risk of HBV infection because of very low vaccine uptake and poor post-exposure practices. Their knowledge of HBV infection is non-optimal, with lack of information and support regarding HBV vaccination. The prevalence of HBs Ag is high (11 %). There is urgent need for health policies advocating HBV vaccination among health care workers in Cameroon in order to curb the burden and occupational risk of HBV infection in the hospital environment.

## Abbreviations

anti-BHs Ab, hepatitis B surface antibodies; anti-HBc Ab, hepatitis B core antibodies; anti-HBe Ab, hepatitis B e antibodies; EDTA, Ethylene Di-amine Tetra-acetic Acid; HBe Ag, hepatitis B e antigen; HBs Ag, hepatitis B surface antigen; HBV, hepatitis B virus; LMIC, low- and middle-income countries
